# Supplementary material for: Dynamic DNA Methylation Changes in the COMT Gene Promoter Region in Response to Mental Stress and Its Modulation by Transcranial Direct Current Stimulation
Source: Biomolecules. 2021 Nov 19;11(11):1726. doi: 10.3390/biom11111726 (PMC8615564; doi:10.3390/biom11111726)
Supplement: Supplementary file 1 [file biomolecules-11-01726-s001.zip › biomolecules-1450310-supplementary.pdf]

Supplementary Material

Data from pilot cohort only (n = 22)

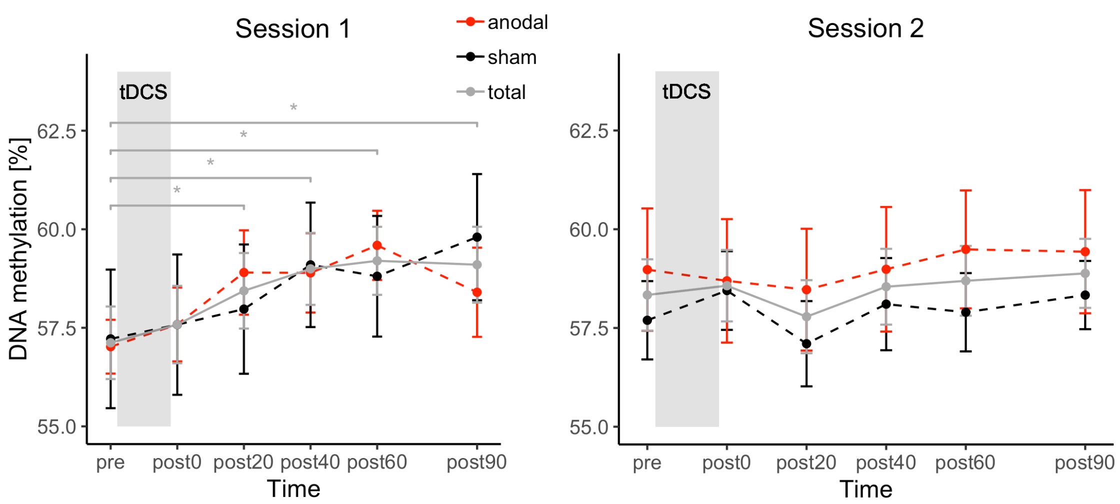

Supplementary Figure S1. DNA methylation changes in pilot cohort during each session with regard to stimulation condition. % DNA methylation is shown separately for the six time points during each session. Each participant in the anodal stimulation group in session 1 (n = 11) was receiving sham stimulation in session 2, and vice versa (n = 11). Error bars depict standard errors of the mean; asterisks mark $p < 0.05$.

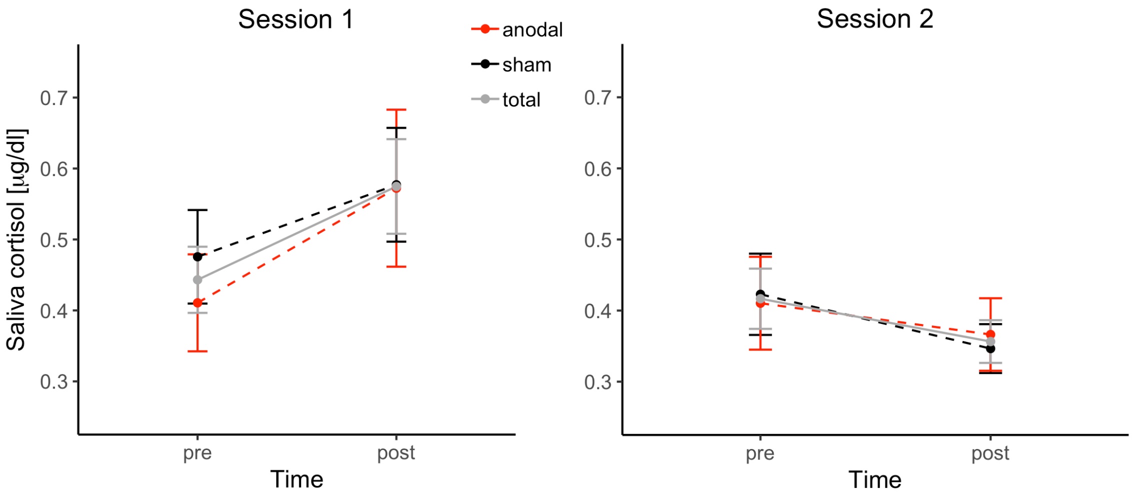

Supplementary Figure S2. Cortisol concentration changes in pilot cohort during each session with regard to stimulation condition. Saliva cortisol levels are shown separately for each session in pre- and post-task condition. As the order of received stimulation (anodal/sham or sham/anodal) was a between-subject factor, participants receiving anodal stimulation during the first session (n = 11) received sham stimulation during their second session, and vice versa (n = 11). Error bars depict standard errors of the mean.

Data from replication cohort only (n = 20)

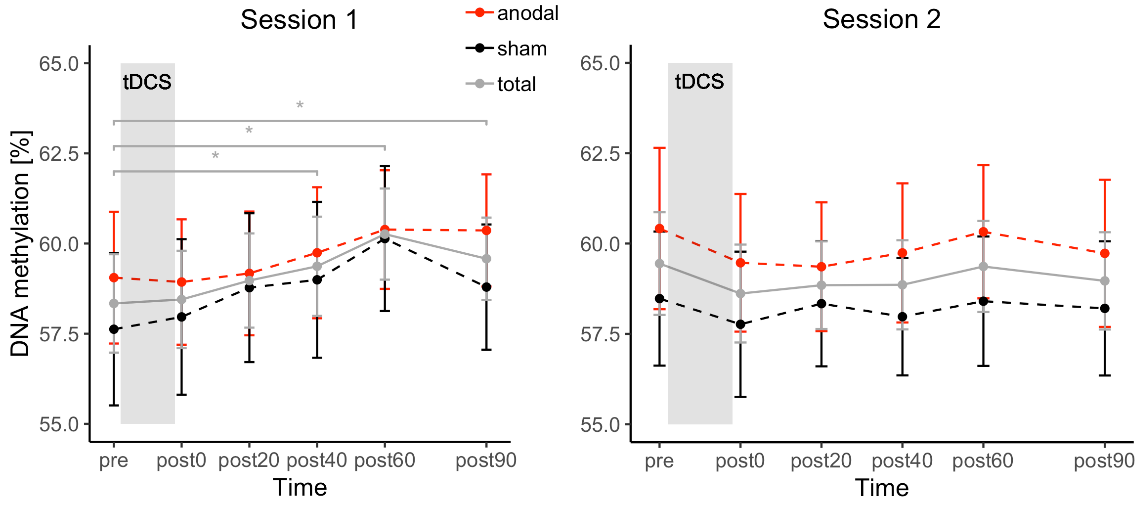

Supplementary Figure S3. DNA methylation changes in replication cohort during each session with regard to stimulation condition. % DNA methylation is shown separately for the six time points during each session. Each participant in the anodal stimulation group in session 1 (n = 10) was receiving sham stimulation in session 2, and vice versa (n = 10). Error bars depict standard errors of the mean; asterisks mark $p < 0.05$.

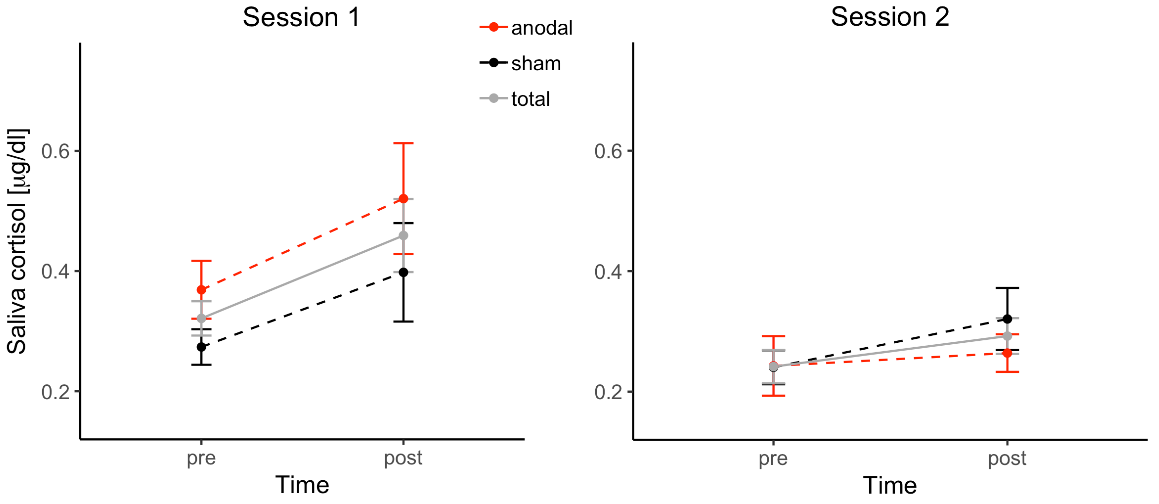

Supplementary Figure S4. Cortisol concentration changes in replication cohort during each session with regard to stimulation condition. Saliva cortisol levels are shown separately for each session in pre- and post-task condition. As the order of received stimulation (anodal/sham or sham/anodal) was a between-subject factor, participants receiving anodal stimulation during the first session (n = 10) received sham stimulation during their second session, and vice versa (n = 10). Error bars depict standard errors of the mean.

Supplementary Table S1. tDCS adverse effects. Adverse sensations were assessed on a 5-point Likert scale (n = 42). A nominal significant difference was observed for a tingling sensation at the site of the electrode. However, this difference did not remain significant when correcting for multiple testing.

Sensation	Sham tDCS Mean (SD)	Anodal tDCS Mean (SD)	*t*	*P*
Tingling at the site of the electrode	2.17 (1.20)	2.71 (1.35)	2.31	0.026
Tingling elsewhere in the area of the head	1.22 (0.52)	1.41 (0.92)	1.19	0.243
Exhaustion	1.07 (0.26)	1.20 (0.46)	1.40	0.168
Slight itching	1.68 (0.99)	2.05 (1.22)	1.78	0.083
Headache	1.05 (0.22)	1.05 (0.32)	0.00	1.000
Nausea	1.00 (0.00)	1.00 (0.00)	-	-

Furthermore, we asked participants at the end of both sessions for their subjective assessment of whether they received sham or verum stimulation. No significant differences in subjective assessments were found between sham and verum conditions (Fisher's exact test: *p*=0.758 (session 1), *p*=0.058 (session 2)).

Experimental Procedure

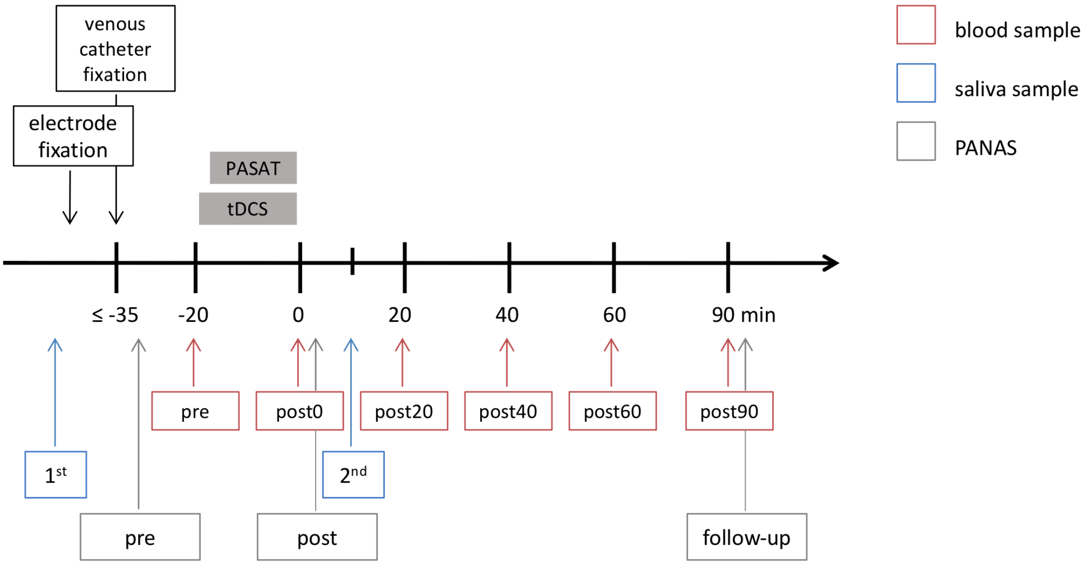

Supplementary Figure S5. Experimental procedure. Schematic representation of an experimental session.

Analysis of Variance Tables

Supplementary Table S2. Task performance

**Term**	***F***	***p***
**Intercept**	$F(1,202) = 776.10$	$p < 0.001$
**Stimulation**	$F(1,202) = 0.17$	$p = 0.68$
**Session**	$F(1,202) = 142.71$	$p < 0.001$
**Task block**	$F(2,202) = 5.40$	$p = 0.005$
**Stimulation * task block**	$F(2,202) = 0.83$	$p = 0.44$
**Session * task block**	$F(2,202) = 2.33$	$p = 0.10$

Supplementary Table S3. Affect changes

**Term**	***F***	***p***
**Intercept**	$F(1,199) = 5366.20$	$p < 0.001$
**Stimulation**	$F(1,199) = 0.11$	$p = 0.75$
**Session**	$F(1,199) = 2.75$	$p = 0.09$
**Time**	$F(2,202) = 11.02$	$p < 0.001$
**Stimulation * session**	$F(1,199) = 0.28$	$p = 0.60$
**Stimulation * time**	$F(2,202) = 1.17$	$p = 0.31$
**Session * time**	$F(2,199) = 7.18$	$p = 0.001$
**Stimulation * session * time**	$F(2,199) = 1.72$	$p = 0.18$

Supplementary Table S4. DNA methylation changes

**Term**	***F***	***p***
**Intercept**	$F(1,457) = 3876.71$	$p < 0.001$
**Stimulation**	$F(1,457) = 5.89$	$p = 0.016$
**Session**	$F(1,457) = 0.41$	$p = 0.52$
**Time**	$F(1,457) = 17.09$	$p < 0.001$
**Stimulation * time**	$F(1,457) = 0.14$	$p = 0.71$
**Session * time**	$F(1,457) = 14.25$	$p < 0.001$

Supplementary Table S5. Cortisol concentration changes

Term	*F*	*p*
**Intercept**	$F(1,121) = 6.33$	$p = 0.013$
**Stimulation**	$F(1,121) = 2.51$	$p = 0.12$
**Session**	$F(1,121) = 6.12$	$p = 0.014$
**Time**	$F(1,121) = 0.38$	$p = 0.54$
**Stimulation * time**	$F(1,121) = 0.01$	$p = 0.93$
**Session * time**	$F(1,121) = 6.75$	$p = 0.011$

Sample description

Supplementary Table S6. Sample characteristics

	**Group sham – anodal (n=21)**	**Group anodal – sham (n=21)**
**Age [years]**	22.71 (± 2.81; 18-28)	24.19 (± 3.44; 18-29)
**Years of education [years]**	16.38 (± 2.99; 12-22)	17.40 (± 3.29; 12-23)
**Math performance at school***	10.76 (± 2.47; 5-15)	10.23 (± 2.93; 5-15)
**Laterality index****	99.05 (± 4.37; 80-100)	97.08 (± 8.00; 70-100)
**BMI [kg/m^2]**	23.00 (± 2.10; 19.59-27.44)	22.86 (± 1.83; 19.94-25.98)
**SCL-90-R**		
Somatization	0.16 (± 0.18; 0.00-0.58)	0.21 (± 0.22; 0.00-0.75)
Obsessive-compulsive	0.41 (± 0.36; 0.00-1.20)	0.38 (± 0.29; 0.00-1.00)
Interpersonal sensitivity	0.18 (± 0.19; 0.00-0.67)	0.22 (± 0.28; 0.00-1.00)
Depression	0.19 (± 0.22; 0.00-0.69)	0.27 (± 0.27; 0.00-1.00)
Anxiety	0.18 (± 0.13; 0.00-0.40)	0.11 (± 0.13; 0.00-0.40)
Anger-hostility	0.19 (± 0.22; 0.00-0.83)	0.21 (± 0.26; 0.00-0.83)
Phobic anxiety	0.05 (± 0.12; 0.00-0.43)	0.01 (± 0.03; 0.00-0.14)
Paranoid ideation	0.12 (± 0.21; 0.00-0.83)	0.20 (± 0.33; 0.00-1.17)
Psychoticism	0.06 (± 0.09; 0.00-0.30)	0.15 (± 0.21; 0.00-0.70)
GSI	0.18 (± 0.12; 0.00-0.44)	0.21 (± 0.18; 0.00-0.69)
***COMT* genotype**		
Val/Val	7	7
Val/Met	8	9
Met/Met	6	5

Mean (± standard deviation, range), GSI: Global severity index, SCL-90-R: Symptom-Checklist-90-Revised

* According to the German academic grading system (15-point scale)

** Edinburgh Inventory[1]

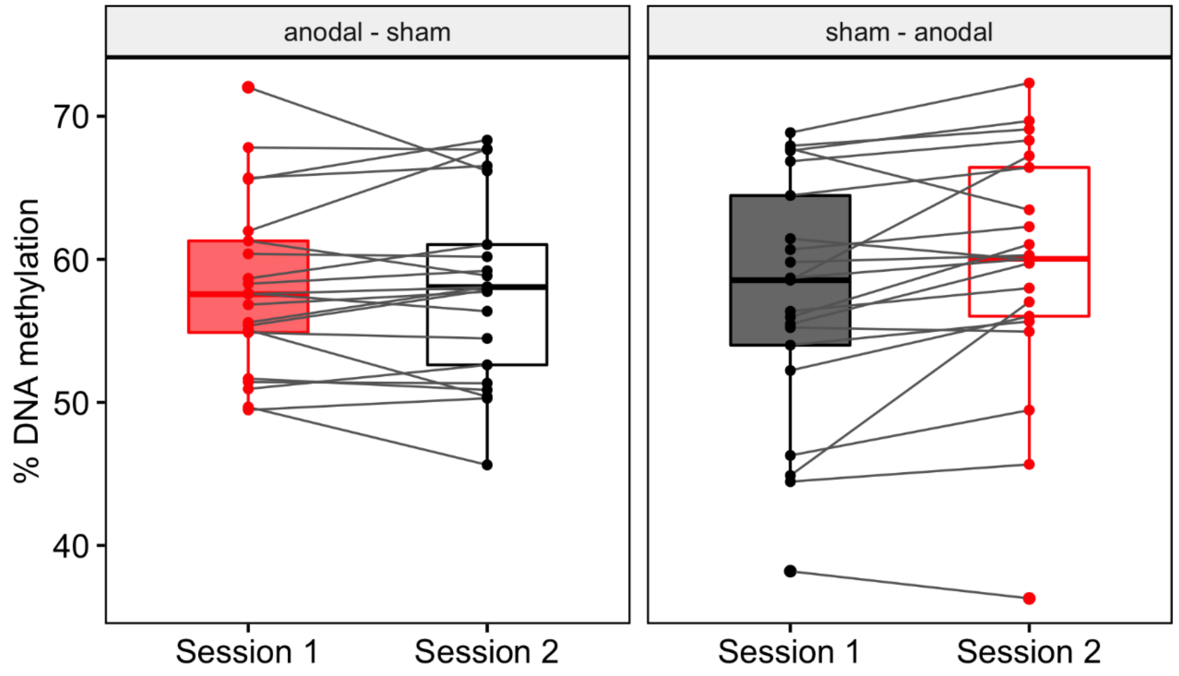

Supplementary Figure S6. Preservation of DNAm changes over one week with regard to the order of stimulation condition. % DNAm for session 1 and 2 at time point 'pre' grouped by order of stimulation conditions ('anodal - sham' (n = 21) or 'sham - anodal' (n = 21)). The figure illustrates the comparison of % DNAm before ('pre') the first (session 1) and second (session 2) PASAT training within subjects who received tDCS ('anodal - sham') and subjects who did not receive effective tDCS in session 1 ('sham - anodal').

References

1 Oldfield, R. C. The assessment and analysis of handedness: the Edinburgh inventory. *Neuropsychologia* **9**, 97-113 (1971).
